# Bioelectric Activity in the Suprachiasmatic Nucleus—Pineal Gland System in Children with Adolescent Idiopathic Scoliosis

**DOI:** 10.5402/2012/987095

**Published:** 2012-08-28

**Authors:** Dmitry Yu Pinchuk, Sergey S. Bekshaev, Svetlana A. Bumakova, Mikhail G. Dudin, Olga D. Pinchuk

**Affiliations:** ^1^Human Brain Research Centre, Finlandsky pr. 4-A, Office 901, St. Petersburg 194044, Russia; ^2^Ogonyok Rehabilitation Center of Pediatric Orthopedics and Traumatology, St. Petersburg Highway 101, St. Petersburg 198515, Russia; ^3^Sechenov Institute of Evolutionary Physiology and Biochemistry, Russian Academy of Sciences, Toreza st., 44, St. Petersburg 194223, Russia

## Abstract

The purpose of this work is to identify a role of the pineal gland/suprachiasmatic nucleus system in adolescent idiopathic scoliosis (AIS) aetiology and pathogenesis. To analyze electroencephalograms of 292 children with AIS and in 46 healthy subjects, a processing method was used to assess three-dimensional coordinates of electric equivalent dipole sources (EEDSs) within the brain. Amounts of EEDSs in the pineal gland and suprachiasmatic nucleus (SCN) area were assessed in different age groups and during the progress of orthopaedic pathology. It was shown that children with AIS, compared with healthy children, were characterized by a higher level of electric activity (as judged by EEDS values) in the pineal gland area. It was also revealed that the number of EEDS in the pineal gland area increases significantly with increased severity of spinal deformation, while their number in the suprachiasmatic nucleus (SCN) area decreases compared with the number in healthy peers. Changes in electric activity and changes in the pineal gland and SCN area suggest that mechanisms of AIS aetiology and pathogenesis involve functional disturbances in brain areas responsible for the formation and maintenance of normal biorhythms, including osteogenesis and bone growth.

## 1. Background

Adolescent idiopathic scoliosis (AIS) is a disease of unknown aetiology, characterized by a complex three-dimensional deformation of the spinal column as the most common paediatric orthopaedic pathology. Its incidence, according to different sources, varies from 2 to 6% of the total paediatric population, while 3 in 1000 adolescents require severe reconstructive spinal surgery [1]. AIS is characterized by its relationship with the pubertal period, its much higher incidence in adolescent girls as compared with boys (from 1 : 7 to 1 : 10 according to various authors), and poor prediction of the course of the disease. The process of deformation can stop spontaneously without treatment at some point, but it can also progress rapidly up to an angle of 70 degrees or more [1, 2]. Although the first mention of scoliosis goes back as far as Hippocrates (c. 460–370 BC), to date there is no factual, scientifically feasible theory of AIS aetiology and pathogenesis [3]. Accordingly, there are no reliable methods for predicting the extent and rate of spinal deformation, nor for its effective treatment and prevention. 

One of the main reasons for this state is the lack of an effective model reproducing all the specific features of the pathological process in animals. The disease therefore is difficult to study. Conventional animal models (peripheral nerve disruption, muscle extirpation, vertebral and intertverebral disc lesions, etc.) are artificial to a considerable extent, so that their results cannot be satisfactorily transferred into clinical practice [3]. 

The most effective scoliosis models are those of pinealectomized chickens [4] and bipedal infant rats [5]. It was shown that removal of the pineal gland in chickens leads to stage-by-stage three-dimensional deformation of the spinal column, as in humans. The authors interpreted development of experimental scoliosis as a result of disturbance of the symmetrical growth of spine and paraspinal muscles, due to disturbance of melatonin synthesis [4]. The same results were obtained in experiments on rats, which, at the birth of resected front paws and tail, caused the animal to move on two hind legs (bipedal rats). Thus, researchers have obtained a model of a mammal with the vertical spinal column. Pinealectomy in these rats also causes scoliosis [5]. However, the mechanism of this deformation in experimental model is still disputable [6–8]. 

In some experimental work administration of the hormone melatonin, normally secreted by the pineal gland, to pinealectomized animals was shown to prevent deformation [9]; while in other works this claim is disputed [10]. Similar ambiguity is observed concerning children with AIS. A decrease in melatonin concentration was reported in the blood of children with AIS [7]; however, an attempt to administer melatonin to such children (a sort of substitution therapy) was ineffective. Other authors [11] found no significant differences in blood concentrations of this hormone and melatonin metabolites in the urine of healthy children compared with that of children with AIS. Positron emission tomography (PET) studies also failed to reveal any differences in metabolic intensity in a pineal gland area in patients with AIS compared with that of healthy controls [12]. Thus, the only evidence is that the removal of the melatonin-producing pineal gland with the resulting failure of melatonin production in growing animals with a vertical spine leads to scoliosis similar to that observed in humans. Muscle tone changes due to disrupted sleep-wake cycles or homeostasis [4, 13] are considered by the majority of specialists to be entirely irrelevant to these phenomena [14]. 

The main objective of the present work was to study the functioning of brain structures which regulate melatonin secretion in patients with AIS, using electrophysiology methods in order to better understand AIS aetiopathogenesis and possibly to develop methods for the prevention, treatment, and prognosis of the deformation process.

## 2. Methods

The study included 338 children and adolescents aged from 8 to 16 years. The main group contained 292 children with AIS of varied severity (44 children, Grade I; 109 children, Grade II; 105, Grade III; 34, Grade IV), and the control group consisted of 46 healthy children of the same age group. All were patients at the Ogonyok Rehabilitation Center of Pediatric Orthopedics and Traumatology during 2006–2009. The study was approved by the Institutional Ethics Committee of the Ogonyok Rehabilitation Center of Pediatric Orthopedics and Traumatology. The extent of deformation during and after the treatment is in accordance with the V. D. Chaklin X-ray grading system accepted in Russia [15]. According to this system, Grade I represents scoliotic deformation from 1 to 10° by Cobb, Grade II from 11 to 25°, Grade III from 26 to 50°, and Grade IV >50°. 

According to the pattern of deformation progress, patients were assigned to nonprogressive, progressive, and rapidly progressive AIS forms. Patients with radiologically detected spinal deformation increase during a previous year of ≤5° by Cobb were defined as nonprogressive forms, patients with an annual deformation > 5° ≤ 10° were defined as progressive, and patients with annual deformation ≥10° by Cobb were defined as rapidly progressive AIS forms—see Table 1.

EEGs were recorded on a Mitsar 21EEG electroencephalography system (Russia) simultaneously and unipolarly from 21 channels according to the international 10–20 system relative to a shorted reference earlobe electrode. The frequency of data points was 250 Hz. EEG recording was performed on all subjects at the same time (from 11 AM to 1 PM) in a state of quiet wakefulness with closed eyes. The recording areas of 80 sec duration without artifacts were used for further processing. It is known that sudden light exposure during one of the “dark” periods blocks melatonin secretion; however, there was no reaction during conventional daylight periods when light was suddenly switched off [16]. That is why EEG from recording subjects with closed eyes in quiet wakefulness during daytime is methodically reasonable. 

EEG processing was performed using the 3DLocEEG software [17] developed in In Vitro [18]. This software calculates equivalent dipole source coordinates (hereafter referred to simply as dipoles) from EEGs. The algorithm is based on solving a reverse electroencephalography task and is limited to a mathematical procedure of detecting sources of electrical activity, which are located in a three-dimensional brain space, from integral scalp-recorded potentials (EEG) [19]. Lately, a method of three-dimensional localization of EEG dipole sources is being successfully used in both applied and fundamental researches [20, 21]. 

In the 3DLocEEG program, a model of a mobile equivalent electric dipole in an isotropic space is used. An algorithm for minimizing the non-dipole component in the multipole decomposition of potentials is implemented. This algorithm was proposed to solve the task of three-dimensional localization of dipole sources in electrocardiograms [22]. 

In analysing each of digitized EEG, the program calculates the coordinates of an equivalent electric dipole according to the algorithm specified. The path of the point with the derived coordinates is imaged and superimposed on anatomical brain structures according to the atlas by Mai et al. [23]. In this atlas, a system of intracerebral coordinates by Talairach and Tournoux is used [24]. 

In our case, the points are reflected on brain sections (sagittal, horizontal, and frontal) of 1 cm thickness (a total of 36 sections). Sections are scaled up by individual patient's skull sizes according to recommendations [23]. For this, measurements of the distance between skull reference points (Inion-Nasion, between external ear canals, etc.) and the distance between electrodes (a total of 32 measurements) are required. By means of such data, three-dimensional coordinates of recording electrodes are determined and are then used for approximation of the skull surface. 

We used a program with a digitizing frequency of 250 Hz, thus obtaining a new point with coordinates of a moving dipole every 4 msec. When all dipole coordinates along with their “powers” (values proportional to a dipole moment vector length) are known, points with maximal power are found on a previously chosen temporal EEG segment. Points with minimal power are removed from sections by varying the observed/highest threshold ratio; generally (but not always), these are the noise points due to the impossibility of entirely eliminating artifacts of various kinds during EEG recording. Only dipoles with coordinates within the pineal gland and suprachiasmatic nucleus (SCN) were analyzed. The number of dipoles in these areas was standardized relative to a total number of recorded dipoles. It is important to note that pineal gland neurons (pinealocytes) together with the capability to secrete peptide hormones (neuropeptides) specific to endocrine cells maintain their neuron organization and function, that is, are able to generate and disseminate nerve pulses [25].

The data were processed in Statistica (StatSoft V. 6.1) using Mann-Whitney  *U*-test for independent samples.

The study was approved by the Institutional Ethics Committee of the Ogonyok Rehabilitation Center of Pediatric Orthopedics and Traumatology.

## 3. Results

**Figure 1:**
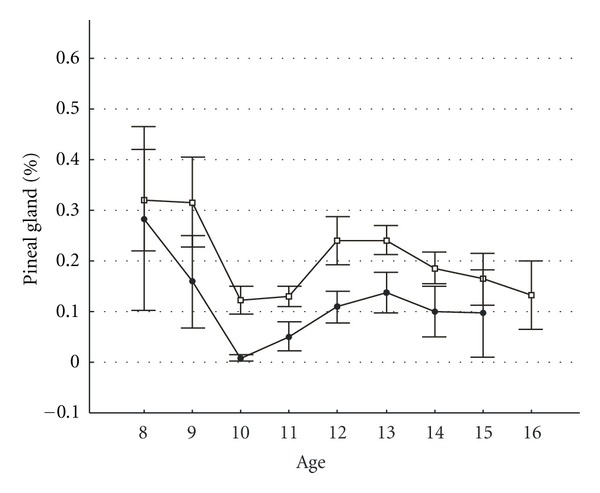
Age-specific changes in EEDS activity in the pineal gland area in children with idiopathic scoliosis compared with those of healthy subjects. The  *y*-axis shows mean number of dipole sources in the pineal gland area (% of total recorded). The  *x*-axis shows age in years. White boxes represent patients with idiopathic scoliosis. Black points represent healthy subjects. Vertical bars denote 0.95 confidence intervals.

To study age-specific changes in bioelectric activity (BA) of the pineal gland, EEGs from children aged from 8 to 16 years were analyzed in groups of healthy subjects and in patients with AIS. Dipole amounts in the pineal gland area (% of total recorded dipoles) averaged by different ages in healthy children, and children with AIS are presented in Figure 1 as M ± 1.96 SD. 

Statistically significant differences (*P* < 0.05 by Mann-Witney test) between patients with AIS and healthy peers are found in age groups of 10–14 years (i.e., in the pubertal period). In general, dipole numbers in the pineal gland area were lower in healthy subjects than in AIS patients, even in those age groups where these differences were statistically not significant. Adolescent girls strongly predominated among children with AIS, and their typical pubertal age was from 10 to 14 years; thus, EEGs of girls of this age were selected for analysis of BA activity in the pineal and SCN areas.

**Figure 2:**
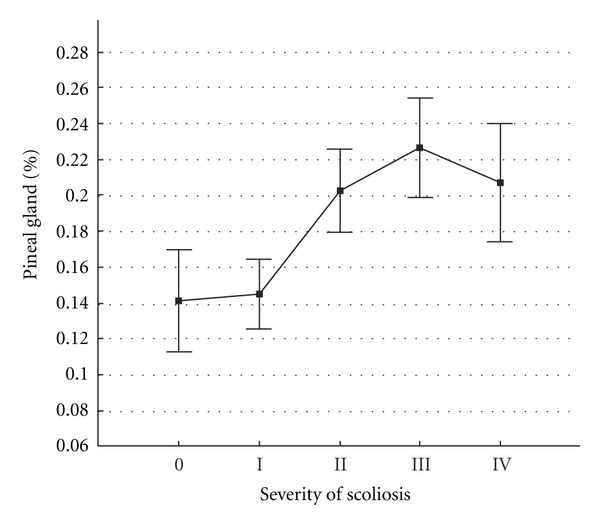
Levels of EEDS activity in the pineal gland area in patients with scoliotic spine deformation of varied severity and in healthy subjects. The  *y*-axis shows mean number of dipole sources in the pineal gland area (% of total recorded). The  *x*-axis shows (0) healthy subjects and patients with different grades (I, II, III, IV) of idiopathic scoliosis severity. Vertical bars denote 0.95 confidence intervals.

The distribution in the number of dipoles (as % of total number of recorded dipoles) in the pineal gland area in patients with different AIS severity and healthy subjects is presented in Figure 2.

**Figure 3:**
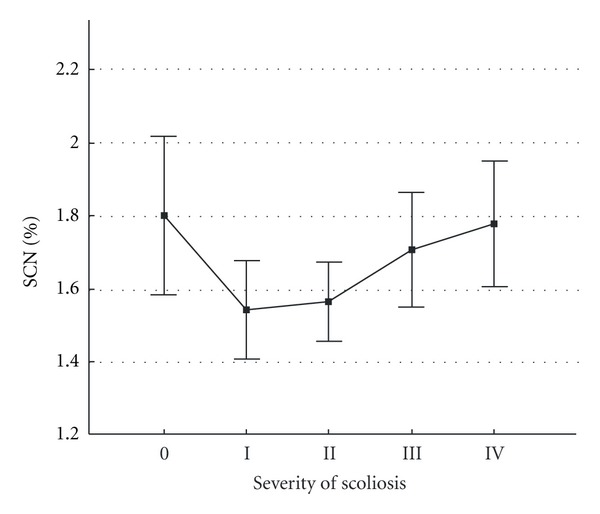
Levels of EEDS activity in the suprachiasmatic nucleus (SCN) area in patients with different grades of scoliotic spine deformation and in healthy subjects. The  *y*-axis shows mean number of dipole sources in suprachiasmatic nucleus (SCN) area (% of total recorded). The *x*-axis shows (0) healthy subjects and patients with different grades (I, II, III, IV) of idiopathic scoliosis severity. Vertical bars denote 0.95 confidence intervals.

The figure shows that the lowest activity in the pineal gland was recorded in healthy subjects. In patients with AIS the number progressively increased with worsening of the condition, achieved maximal values in patients with Grade III severity, and slightly decreased in patients with Grade IV. Among female patients with Grade IV, girls aged 14 years predominated (57% of cases); in this age group, the growth process and consequently the progress of deformation already slow down. Instead, the total number of dipole sources in the right and left SCN areas was inversely proportional (Figure 3)—the greatest activity in the SCN area was noted in healthy subjects. It gradually decreased with AIS progress the worsening of the clinical condition worsening and again slightly increased in patients with Grade III and IV AIS. 

Differences in dipole quantities between healthy subjects and patients with different grades of AIS severity are not statistically significant and in this case one can speak of an observed trend only. The most likely reason for the lack of significant differences is a limited resolution of the method used and significantly smaller SCN sizes compared to the size of the pineal gland. Thus, considerably more dipoles reflecting activity of adjacent brain structures were registered in the SCN compared to the pineal area. 

**Figure 4:**
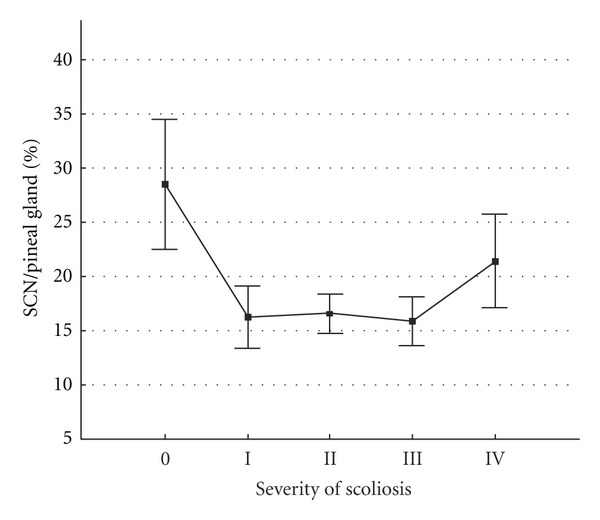
Ratio of EEDS activity level in the suprachiasmatic nucleus (SCN) to number of equivalent dipole sources in the pineal gland area. The  *x*-axis shows (0) healthy children and children with different grades (I, II, III, IV) of idiopathic scoliosis severity. The  *y*-axis shows ratios of the number of dipole sources in the suprachiasmatic nucleus (SCN) area to the number of equivalent dipole sources in the pineal gland area. Vertical bars denote 0.95 confidence intervals.

The ratio of SCN/pineal dipole quantity, in our opinion, may be an integral bioelectric characteristic of the relationship in the “pineal gland—SCN” system and is presented in Figure 4.

The observed statistically significant differences suggest differences in the functioning of the pineal gland—SCN system between healthy subjects and patients with AIS of varied severity. 

Research of the pineal gland—SCN system functioning in AIS patients not only with varied spine deformation severity but also with varied progressive activity deserves special attention. Bioelectric processes in central nervous system (CNS) reflecting abnormalities in the normal pubertal period often stop by the time of radiological documentation of spinal curvature. In many cases, EEGs of patients with Grade IV AIS and marked spinal deformation hardly differ from those of healthy peers. 

**Figure 5:**
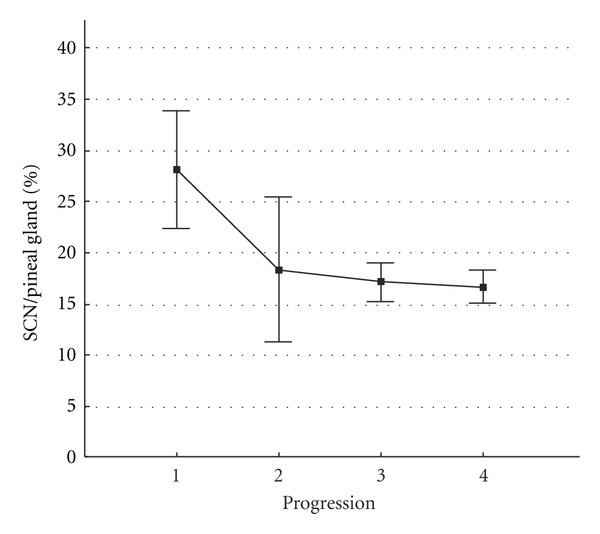
Comparison of ratios of EEDS activity levels in the suprachiasmatic nucleus (SCN) area to the number of equivalent dipole sources in the pineal gland area in groups of healthy children and patients with different time courses of idiopathic scoliosis. The  *y*-axis shows ratios of dipole source numbers in the suprachiasmatic nucleus (SCN) area to numbers of equivalent dipole sources in the pineal gland area. The  *x*-axis shows groups of patients with different time courses of idiopathic scoliosis: (1) healthy subjects, (2) nonprogressive forms, (3) progressive forms, (4) rapidly progressive forms. Vertical bars denote 0.95 confidence intervals.

In Figure 5, ratios of SCN to pineal gland dipole numbers are provided in groups of healthy subjects and patients with various progression patterns.

As it is evident from the figure, this parameter decreased from healthy patients to patients with nonprogressive AIS and achieved statistically significant differences between healthy children and patients with progressive and rapidly progressive forms.

## 4. Discussion

In healthy children, blood melatonin concentration increases gradually up to one year and is maintained on a relatively high level up to the pubertal period. The major role of melatonin in this age period is to prolong the sleeping period and to suppress sex hormone secretion. In puberty, the amount of circulating melatonin decreases; moreover, this decrease is more prominent when sexual maturity (pubertal period) begins. Our results on age-specific dipole distribution in the pineal gland area of healthy subjects confirm the established fact of an age-related decrease in pineal gland activity [26, 27]; hence, our chosen research method is appropriate. Besides the pubertal period coincides with a period of the most rapid progress of spinal deformation in children with AIS. In this period, an amount of serum melatonin decreases by 4 times. 

Normally, the pubertal period begins after achieving appropriate body maturity. The triggering of the pubertal phase is considered to be a result of concordance of two physiological processes in the hypothalamus: luteinizing hormone releasing hormone (gonadotropin-releasing hormone) production by the arcuate nucleus and activity of the SCN as a circadian rhythm regulator. Slowed frequency of SCN oscillations leads to increased stimulation of gonadotropin-releasing hormone secretion. As a result, gonadotropin hormones (GTHs) are episodically released during the early pubertal period, particularly luteinizing and follicle-stimulating hormones during nighttime. Then, rhythmic GTH secretion is achieved which stimulates synthesis and secretion of sex hormones by gonads, along with gametogenesis. In experiments on animals it was established that reproductive function is regulated not by the immediate influence of the pineal gland on sex glands but by the modulation of hypothalamic-hypophysis system activity (preoptic area of the hypothalamus and the tuberal part of the hypophysis). Inhibiting influence of the pineal gland on sexual functions is mediated by some factors: melatonin reduces or inhibits secretion of gonadotropin-releasing hormone by the hypothalamus and secretion of gonadotropins by the anterior lobe of the hypophysis. At the same time, pinealocytes produce the antigonadotropin hormone that reduces secretion of luteinizing hormone-releasing hormone by the anterior lobe of the hypophysis [27]. 

It may be supposed that differences in the functioning of this system according to electrophysiology BA tests in the SCN and pineal areas between healthy subjects and patients with AIS are bioelectric reflections of their own and of downstream functional disorders. One of these disorders apparently leads to delayed sexual maturity in girls with AIS, which was demonstrated in some anatomic and physiologic studies [28]. 

However, this retardation is quite small, within 0.5–1 year. Clinically relevant retardation of sexual maturity is determined (by Tanner) as delay of sexual maturity signs by more than 2 years from an appropriate mean population age. Thus, deviations in sexual maturity mechanisms in AIS are much more delicate than those considered in clinical endocrinology in cases of quite severe disorders of sexual development [27]. It should be noted that there is no generally accepted opinion on delayed sexual development in girls with AIS. Some authors, on the contrary, report an accelerated sexual development in girls with AIS [29]. However, quite mild delay of sexual maturity by itself does not explain scoliotic deformation in children with AIS. It more likely indicates disorders in pineal gland/SCN system activity. The possible reasons for explaining the development of spinal deformation in children may be obtained in considering this issue from a position of chronobiology. 

The pineal gland/SCN system is the main synchronizer of biologic rhythms of the body, effecting their coordination [30]. Defects in the activity of this system (abnormal melatonin production and/or shift of its secretion phase) result in desynchronization of body biorhythms and the development of various pathologies. An inverse pineal gland relationship with SCN plays a major role here, as its disturbance changes periods of internal pacemaker activity, thus leading to desynchronization and impairment of related body processes. In the case of pineal gland/SCN system dysfunction, disorders of the hypothalamopituitary-adrenal axis are observed, particularly in the secretion of cortisol and other mineral corticoids, and the osteoblast function [30–32]. This results in multiple disruptions of various functional body systems [30]. In vertebrate animals, somatotropic hormone (STH), or growth hormone, of the anterior lobe of the hypophysis is the main regulator of protein synthesis, which also affects mineral, carbohydrate, and lipid metabolism. Although these hormone effects on tissues are mediated by somatomedins, there is a specific relationship between growth rhythms and periodic changes in STH secretion and inflow into tissues. STH secretion coincides with slow-wave sleep and mainly takes place during the first half of the night, that is, STH secretion is also affected by the SCN/pineal gland system [25, 30]. 

During the years 1960–1980 it was shown that changes in illumination patterns resulting in abnormal SCN/pineal gland system activity led to marked osteosynthesis disturbance, up to stopping bone tissue growth [33, 34]. In one of the recent works [35] dedicated to AIS aetiology and pathogenesis it was also shown that changes in illumination patterns led to scoliosis development in 15% of chickens with an intact pineal gland. However, in this case serum melatonin decreases almost down to zero, as opposed to children with AIS. 

If pineal gland BA is considered as the bioelectric reflection of its secretory activity, then our data on increased pineal gland BA in children with AIS compared to healthy peers are in conflict with those obtained in experiments with pinealectomized animals; some clinical trials suggesting decreased serum melatonin in cases of development of spinal scoliosis. On the other hand, our data support the data of most clinical works suggesting the normal (more frequently) or even increased levels of melatonin in patients with AIS. 

In our opinion, the reason for AIS development is probably not decreased (or increased) serum melatonin (which appears to take place in some cases as well), but a disrupted rhythm of its secretion. This leads to discrepancy between delicate by related mechanisms of hormone excretion and hormone sensitivity of body tissues. It is known that maximal and minimal hormone concentrations differ more than 10-fold over a 24-hour period. Therefore, along with hormone concentrations, the temporal coincidence of hormone secretion and maximal reactivity of body tissues (in this case, bone tissue) to their exposure is extremely important. It was demonstrated that in some diseases, such as diabetes mellitus, depressions, Alzheimer's and Parkinson's diseases, immunodeficiency, and cancers, the pineal gland synthesizes less melatonin or, more importantly, disorders of melatonin secretion rhythm occur, while the total amount of synthesized melatonin is maintained [29]. However, virtually all clinical studies of pineal gland/SCN system in AIS patients assess mean daily/nightly concentrations of serum melatonin and its urine metabolites; occasionally, few discrete samples are taken. These methods may not be capable of assessing delicate mechanisms of temporal disorders in SCN/pineal gland activity. 

Dysfunction of pineal gland activity (with SCN as a pacemaker) leads to a discrepancy between related mechanisms of excretion and reception of different hormones (including osteotropic hormones, STH, cortisol) which results in the development of scoliosis on a pinealectomized chicken model. Probably, this mechanism works in humans as well. Moreover, the total melatonin concentration may not decrease during AIS development, imbalanced SCN/pineal gland activities are a sufficient cause. This latter statement may be confirmed by evidence that disruption of pineal conduction paths integrity is enough for scoliosis development on a pinealectomized chicken model, even if pineal anatomy and secretory function are preserved. This surgery is even more effective for experimental scoliosis development than a total pineal gland extirpation [10]. AIS may therefore be referred to the so-called desynchronoses—pathologic conditions associated with biorhythm disorders. 

SCN/pineal gland system dysfunction may be caused by different pathologic factors affecting CNS formation and development in prenatal and early postnatal periods. They include, among others, hormone medications, stress and toxicosis during pregnancy, pathological birth, infections, intoxications, any chronic diseases, and craniocerebral injury. As judged by medical histories of patients under study, pathogenic effects do not correlate with degree of severity. This is probably due to individual limits of functional endurance established during a period of differentiation and development of hypothalamic structures; these limits further characterize a subject's hormonal system throughout his life.

## 5. Conclusions

Changes in electric activity levels in the pineal gland and suprachiasmatic nucleus area revealed in our study suggest that dysfunctions in brain areas responsible for the formation and maintenance of normal biorhythm patterns, including osteogenesis and bone growth, are involved in AIS aetiopathogenesis. Our results may be used for developing methods which are novel and essentially different from those currently used for the treatment of AIS and for preventive measures that are primarily aimed at normalizing the biorhythms of the SCN/pineal gland system.

## Figures and Tables

**Table 1:** Classification of AIS according to the pattern of deformation progress.

AIS form	One-year deformation increase (°Cobb)
Nonprogressive	≤5
Progressive	> 5 ≤ 10
Rapidly progressive	≥10

## Abbreviations

AIS: Adolescent idiopathic scoliosis
EEG: Electroencephalography
EEDS: Electric equivalent dipole sources
SCN: Suprachiasmatic nucleus
BA: Bioelectric activity
GTH: Gonadotropic hormones
STH: Somatotropic hormone.
